# Incident Tuberculosis among Recent US Immigrants and Exogenous Reinfection

**DOI:** 10.3201/eid1105.041107

**Published:** 2005-05

**Authors:** Ted Cohen, Megan Murray

**Affiliations:** * Brigham and Women's Hospital, Boston, Massachusetts, USA;; † Harvard School of Public Health, Boston, Massachusetts, USA;; ‡ Massachusetts General Hospital, Boston, Massachusetts, USA

**Keywords:** Epidemiology, tuberculosis, immigration, re-infection

## Abstract

Mathematical models and molecular epidemiologic investigation support the argument that exogenous reinfection plays an important role in tuberculosis transmission in high-incidence regions. We offer additional data from tuberculosis cases among recent US immigrants which strengthen the claim that reinfection in areas of intense transmission is common.

As the incidence of tuberculosis (TB) in countries with the lowest rates of disease continues to decline, disease in these areas is increasingly borne by immigrants. Recently, researchers have examined patterns of incident disease among persons emigrating from high-incidence (generally >50 cases/100,000 persons/year) to low-incidence regions (generally ≈10 cases/100,000 persons/year) (1–5). While recent immigrants to these countries are at high risk for disease (6), more recent studies also find that elevated TB rates among immigrants to these countries persist for many years after relocation. Most researchers have assumed that TB cases among immigrants from high-incidence areas represent the reactivation of latent infections.

Studies of TB have been conducted to estimate the risk for disease among immigrants, to assess the risk for infection to the native-born population attributable to these immigrants, or to critique existing screening programs. We took an alternate approach and used these data to gain insight into the transmission dynamics that drive the epidemics in the high-incidence areas from which these persons have emigrated. We used data from the US national TB surveillance system to estimate age-specific incidence rates of TB among immigrants by time since arrival in the United States from each of 6 countries of origin: China, Haiti, India, South Korea, Philippines, and Vietnam. We explain that the patterns of incident disease are consistent with the current understanding of the natural history of TB and offer additional support for the argument that infection confers only partial immunity and reinfection plays a major role in areas with high TB incidence.

## The Study

TB cases from all 50 states and the District of Columbia were recorded in the US national TB surveillance system (NTBSS). Report forms collected standard information at the time of diagnosis, including country of origin, age at diagnosis, and years since arrival in the United States (Centers for Disease Control and Prevention, unpub. data). US-born persons were defined as those either born in the United States (or its jurisdictions) or those born in a foreign country who had at least 1 US parent. Those who did not meet these criteria were considered foreign-born persons. Information on immigration status was not collected. Our analyses included all cases reported from 1999 to 2001 among persons who had emigrated from China, Haiti, India, South Korea, Philippines, or Vietnam within 10 years before their diagnosis in the United States. Case reports that were missing information on country of origin were not included in this analysis. While immigrants from Mexico are also at high risk for infection and disease, we excluded cases among this group because the large proportion of unauthorized immigrants precluded a reasonable estimation of population denominators for this subgroup.

We used US immigration statistics to document the number and ages of persons arriving from each of these countries in the years 1989–2001 (Available from http://uscis.gov/graphics/shared/aboutus/statistics/Immigs.htm). To estimate the appropriate population denominators from which these TB cases arose, we applied age-specific mortality rates (categorized as <1–4 years, 5–14 years, 15–24 years, 25–44 years, 45–64 years, and ≥65 years) for US immigrants and assumed that these populations were depleted only by death (no emigration from the United States). Confidence intervals for the country- and age-specific incidence rates were calculated by assuming that TB case counts were Poisson distributed.

## Conclusions

**Figure 1:**
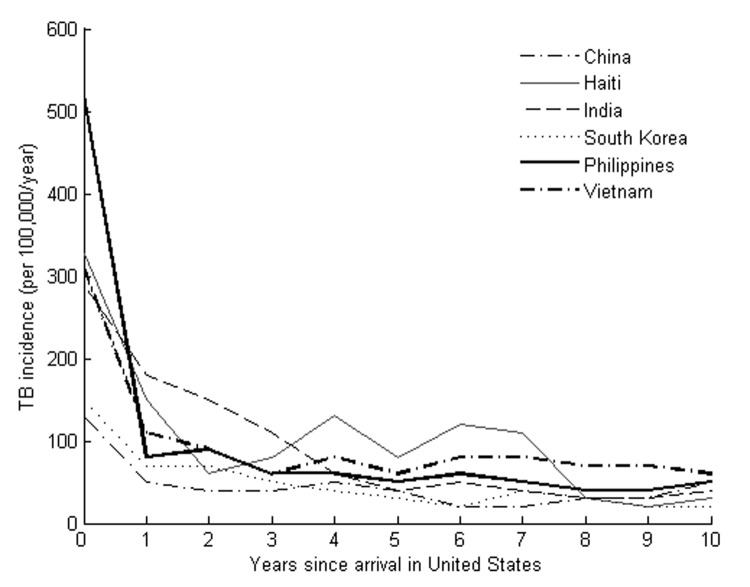
Tuberculosis incidence by time since arrival among recent US immigrants.

From 1999 to 2001, a total of 5,198 cases of TB were recorded in NTBSS among persons who had emigrated from the study countries within the preceding 10 years. Figure 1 depicts the country-specific incidence rate of TB by time since arrival in the United States. Incidence rates among immigrants who have been in the country for <1 year at the time of their diagnosis are similar in relative rank and magnitude to the estimated incidence rates in the country of origin (Table 1) (7). TB incidence rates appear to decrease sharply over the first 2 years in the United States.

**Table 1:** Estimated incidence of tuberculosis among newest immigrants and disease incidence and infection prevalence in the countries of origin

Country	Annual TB incidence in home country (7), cases/100,000 [rank]	TB incidence in first year after arrival in United States, cases/100,000 [rank]	% of population with latent infection in home country (7)
China	113 [5]	130 [6]	36
Haiti	385 [1]	330 [2]	54
India	187 [4]	290 [4]	44
Philippines	314 [2]	520 [1]	47
South Korea	87 [6]	150 [5]	36
Vietnam	189 [3]	310 [3]	44

**Figure 2:**
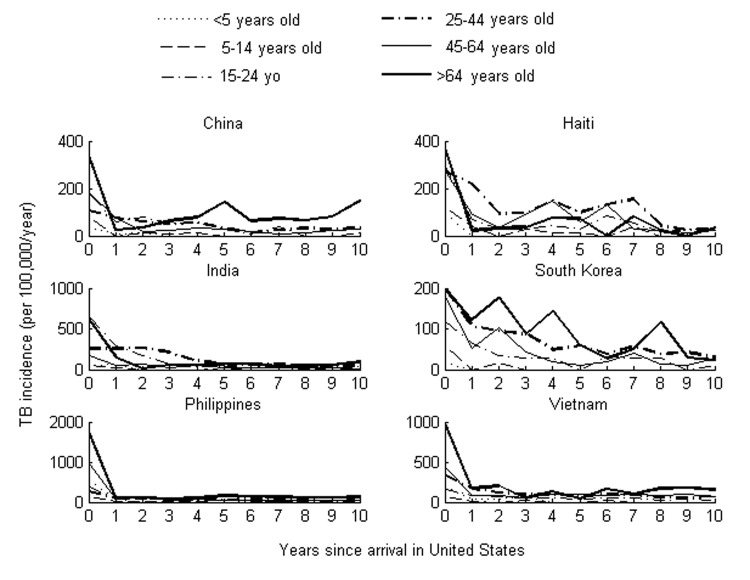
Tuberculosis incidence by time since arrival among recent US immigrants categorized by age group. Note that different scales are used for incidence.

Figure 2 depicts the age-specific incidence rates by time since arrival for US immigrants from each of 6 countries. When incidence rates during the first 2 years were compared with those after 5–10 years in the United States, significantly higher rates were found to occur immediately after arrival in all age groups examined (Table 2). Because incidence rates immediately after arrival will overestimate the true incidence if prevalent cases are misclassified as incident cases, we tested the sensitivity of these findings to the incidence rates calculated for the first 2 years after arrival. We found that this significant elevation in early (<l year after immigration) versus late (years 5–10) incidence persists in almost every age group for all countries even if we assume that we overestimated incidence by a factor of 2 in the early years (Table 2).

**Table 2:** Estimated tuberculosis incidence (cases/100,000) and 95% confidence intervals by time since arrival in United States

Country of origin (age of patient in y)	Time since arrival (y)				
	≤1	2–4	5–10	p value*	p value†
China					
5–14	38 (18–71)	11 (3–25)	0 (0–7)	<0.001	0.008
15–24	119 (81–169)	61 (39–91)	16 (8–29)	<0.001	0.002
25–44	94 (76–115)	55 (43–70)	26 (20–32)	<0.001	0.002
45–64	129 (99–165)	25 (16–39)	18 (13–24)	<0.001	<0.001
≥65	200 (140–276)	61 (36–95)	94 (76–116)	<0.001	0.807
Haiti					
5–14	87 (49–144)	15 (3–43)	10 (1–-35)	<0.001	0.062
15–24	190 (142–247)	32 (16–57)	39 (23–62)	<0.001	0.004
25–44	251 (206–304)	114 (87–147)	62 (50–76)	<0.001	<0.001
45–64	193 (129–277)	93 (55–147)	31 (21–44)	<0.001	0.002
≥65	204 (123–319)	48 (13–122)	30 (12–63)	<0.001	0.002
India					
5–14	44 (21–80)	0 (0–9)	6 (1–18)	0.001	0.085
15–24	485 (415–564)	93 (68–124)	31 (19–46)	<0.001	<0.001
25–44	255 (228–285)	192 (169–217)	55 (47–64)	<0.001	<0.001
45–64	118 (90–152)	44 (31–61)	20 (14–27)	<0.001	<0.001
≥65	384 (285–506)	42 (21–76)	60 (43–83)	<0.001	<0.001
South Korea					
5–14	28 (6–83)	5 (0–26)	0 (0–6)	0.004	0.163
15–24	93 (48–163)	30 (11–66)	15 (6–30)	<0.001	0.050
25–44	157 (120–202)	76 (54–104)	42 (32–55)	<0.001	0.008
45–64	119 (71–185)	51 (28–86)	19 (12–30)	<0.001	0.016
≥65	164 (60–357)	132 (61–251)	48 (27–78)	0.021	0.395
Philippines					
5–14	83 (53–125)	9 (2–22)	2 (0–9)	<0.001	<0.001
15–24	229 (185–280)	59 (42–80)	31 (23–41)	<0.001	<0.001
25–44	195 (168–226)	78 (65–94)	45 (39–52)	<0.001	<0.001
45–64	536 (473–604)	82 (65–102)	48 (40–56)	<0.001	<0.001
≥65	959 (813–1123)	98 (68–137)	128 (107–152)	<0.001	<0.001
Vietnam					
5–14	39 (16–81)	8 (2–23)	4 (1–13)	0.001	0.120
15–24	123 (85–173)	53 (36–75)	40 (31–50)	<0.001	0.158
25–44	261 (222–305)	98 (80–120)	73 (65–82)	<0.001	<0.001
45–64	279 (219–350)	85 (65–109)	85 (73–98)	<0.001	0.009
≥65	592 (417–815)	119 (76–177)	140 (115–168)	<0.001	0.009

*p value comparing the equivalence of incidence rates in ≤1 year to years 5–10. †p value comparing incidence rates after ≤1 year to years 5–10, based on assumption that incidence rate in years ≤1 year was overestimated by a factor of 2.

Numerous studies have documented elevated rates of TB among groups migrating from high-incidence areas to low-incidence areas. Although some researchers have reported an immediate drop in incidence (6), most have found, as we did, that the highest rates of incident disease occur nearest to the time of emigration and that a declining, although still elevated, risk persists for at least a decade. One exception is a recent study from the Netherlands, which reported persistent elevations of incidence for a decade after emigration (4); of note, this study excluded all patients with cases occurring within 6 months of arrival on the basis that these early cases were likely to be prevalent cases.

Peri-immigration stress, malnutrition, and early overdiagnosis have been suggested as potential explanations for the fact that the highest TB incidence rates occur near arrival. The elevated risk that persists for decades has been attributed to the reactivation of latent infections (6), continued transmission among a relatively insular immigrant population (8), and disease secondary to migration back and forth from the country of origin (9). We believe that the pattern of early decline and late stability in incidence seen here and in most studies (and which may also have been seen by Vos et al. had they not excluded the cases occurring in the earliest interval) is consistent with the well-described 2-component phenomenon of disease occurrence after infection. The first phase (lasting ≈1–2 years) is dominated by the rapid progression of recent infections acquired in the country of origin, and the second phase (lasting perhaps decades) is defined by the relatively slow reactivation of latent infections acquired during a person's lifetime before emigration (10). The fact that the oldest immigrants from each country continue to have high incidence of disease 10 years after arrival likely reflects the increased lifetime risk for infection that they acquired while living in the high-incidence region. These dynamics, rather than stress, immune suppression, or malnutrition, may explain the patterns of disease occurrence after arrival.

The steep decline in incident disease among the oldest cohorts within the first 2 years after arrival deserves special attention. While the current incidence of disease in the countries of emigration is very high, the annual risk for infection with TB over the past several decades has steadily declined. Thus, a large proportion of those in the oldest age groups would have been infected with *Mycobacterium tuberculosis* early in their lives. The observed decline in incidence after arrival is strikingly similar to observed patterns of declining risk for disease by time since infection (10) and provides compelling evidence that recent infection in the country of emigration is likely to be responsible for much early incident disease in the oldest age groups. The fact that most of these persons would have also been infected early in their lives argues that exogenous reinfection plays a key role in TB dynamics in high-incidence countries. This conclusion is consistent with other epidemiologic studies that report simultaneous decreases in incident disease across all age groups as the force of infection declines (11); molecular studies that document reinfection in high- (12), moderate- (13), and low-incidence settings (14); and theoretical work that supports the role of reinfection as necessary to explain historical trends of disease (15). Together, these studies argue against the notion that previous infection imparts immunity to future infection and disease. The degree to which natural infection protects a person from subsequent exposures remains an important unanswered question.

Our data support the assertion that persons living in areas with intense transmission are likely to be infected multiple times throughout the course of their lives and, if they progress to disease, are most likely to do so because they were recently infected. Although the importance of exogenous reinfection to TB epidemics has been debated, our data reinforce arguments that reinfection likely plays a major role in high-incidence areas. This finding has numerous implications for designing and evaluating treatment programs in areas of high incidence. For example, in areas of intense transmission, persons receiving treatment who have intermittent or persistent smear-positive sputum may not be treatment "failures" but rather patients with multiple infections. Those who plan empiric treatment strategies for patients with active disease should consider the drug-resistance profiles of currently circulating strains rather than those of strains observed in the past.

## References

[1] Zuber PL, McKenna MT, Binkin NJ, Onorato IM, Castro KG. Long-term risk of tuberculosis among foreign-born persons in the United States. JAMA. 1997;278:304–7. 10.1001/jama.1997.03550040060038 9228436

[2] Talbot EA, Moore M, McCray E, Binkin NJ. Tuberculosis among foreign-born persons in the United States, 1993–1998. JAMA. 2000;284:2894–900. 10.1001/jama.284.22.2894 11147986

[3] Lillebaek T, Andersen AB, Dirksen A, Smith E, Skovgaard LT, Kok-Jensen A. Persistent high incidence of tuberculosis in immigrants in a low-incidence country. Emerg Infect Dis. 2002;8:679–84. 12095434 10.3201/eid0807.010482 PMC2730343

[4] Vos AM, Meima A, Verver S, Looman CW, Bos V, Borgdorff MW, High incidence of pulmonary tuberculosis persists a decade after immigration, the Netherlands. Emerg Infect Dis. 2004;10:736–9. 15200873 10.3201/eid1004.030530 PMC3323101

[5] Watkins RE, Plant AJ. Predicting tuberculosis among migrant groups. Epidemiol Infect. 2002;129:623–8. 10.1017/S0950268802007604 12558347 PMC2869926

[6] Wilcke JT, Poulsen S, Askgaard DS, Enevoldsen HK, Ronne T, Kok-Jensen A. Tuberculosis in a cohort of Vietnamese refugees after arrival in Denmark 1979–1982. Int J Tuberc Lung Dis. 1998;2:219–24. 9526194

[7] Dye C, Scheele S, Dolin P, Pathania V, Raviglione MC. Consensus statement. Global burden of tuberculosis: estimated incidence, prevalence, and mortality by country. WHO Global Surveillance and Monitoring Project. JAMA. 1999;282:677–86. 10.1001/jama.282.7.677 10517722

[8] Kulaga S, Behr M, Nguyen D, Brinkman J, Westley J, Menzies D, Diversity of *Mycobacterium tuberculosis* isolates in an immigrant population: evidence against a founder effect. Am J Epidemiol. 2004;159:507–13. 10.1093/aje/kwh065 14977647

[9] McCarthy OR. Asian immigrant tuberculosis—the effect of visiting Asia. Br J Dis Chest. 1984;78:248–53. 10.1016/0007-0971(84)90136-0 6743520

[10] Sutherland I. The ten-year incidence of clinical tuberculosis following "conversion" in 2550 individuals aged 14 to 19 at the time of conversion. TSRU progress report. The Hague: KNCV; 1968.

[11] Grzybowski S, Styblo K, Dorken E. Tuberculosis in Eskimos. Tubercle. 1976;57(Suppl):S1–15. 10.1016/0041-3879(76)90059-3 797076

[12] van Rie A, Warren R, Richardson M, Victor TC, Gie RP, Enarson DA, Exogenous reinfection as a cause of recurrent tuberculosis after curative treatment. N Engl J Med. 1999;341:1174–9. 10.1056/NEJM199910143411602 10519895

[13] Caminero JA, Pena MJ, Campos-Herrero MI, Rodriguez JC, Afonso O, Martin C, Exogenous reinfection with tuberculosis on a European island with a moderate incidence of disease. Am J Respir Crit Care Med. 2001;163:717–20. 11254530 10.1164/ajrccm.163.3.2003070

[14] Bandera A, Gori A, Catozzi L, Degli Esposti A, Marchetti G, Molteni C, Molecular epidemiology study of exogenous reinfection in an area with a low incidence of tuberculosis. J Clin Microbiol. 2001;39:2213–8. 10.1128/JCM.39.6.2213-2218.2001 11376059 PMC88113

[15] Vynnycky E, Fine PE. The natural history of tuberculosis: the implications of age-dependent risks of disease and the role of reinfection. Epidemiol Infect. 1997;119:183–201. 10.1017/S0950268897007917 9363017 PMC2808840

